# Specific Blood Cells Derived from Pluripotent Stem Cells: An Emerging Field with Great Potential in Clinical Cell Therapy

**DOI:** 10.1155/2021/9919422

**Published:** 2021-08-11

**Authors:** Qian Luo, Honghu Li, Wei Shan, Cong Wei, Yan Long, Shuyang Cai, Xiangjun Zeng, Xue Li, Yulin Xu, Xiaoxiao Xu, Yi Luo, Pengxu Qian, He Huang

**Affiliations:** ^1^BoneMarrow Transplantation Center, The First Affiliated Hospital, School of Medicine, Zhejiang University, Hangzhou, 310012 Zhejiang, China; ^2^Institute of Hematology, Zhejiang University, Hangzhou, 310012 Zhejiang, China; ^3^Zhejiang Engineering Laboratory for Stem cell and Immunotherapy, Hangzhou, 310012 Zhejiang, China; ^4^Zhejiang Laboratory for Systems & Precision Medicine, Zhejiang University Medical Center, Hangzhou, 310012 Zhejiang, China; ^5^Center of Stem Cell and Regenerative Medicine, School of Medicine, Zhejiang University, Hangzhou 310012, China; ^6^Dr. Li Dak Sum & Yip Yio Chin Center for Stem Cell and Regenerative Medicine, Zhejiang University, Hangzhou, 310012 Zhejiang, China

## Abstract

Widely known for self-renewal and multilineage differentiation, stem cells can be differentiated into all specialized tissues and cells in the body. In the past few years, a number of researchers have focused on deriving hematopoietic stem cells (HSCs) from pluripotent stem cells (PSCs) as alternative sources for clinic. Existing findings demonstrated that it is feasible to obtain HSCs and certain mature blood lineages from PSCs, except for several issues to be addressed. This short review outlines the technologies used for hematopoietic differentiation in recent years. In addition, the therapeutic value of PSCs as a potential source of various blood cells is also discussed as well as its challenges and directions in future clinical applications.

## 1. Introduction

It is well known that pluripotent stem cells (PSCs) are composed of induced pluripotent stem cells (iPSCs) and embryonic stem cells (ESCs). In 1981, mouse embryonic stem cells (mESCs) were first obtained from the inner cell mass of early-stage embryos [1]. Similar to mESCs, human ESCs (hESCs) were derived from blastocysts in 1998 [2]. In 2006, Takahashi and Yamanaka successfully produced iPSCs from the embryonic or adult fibroblasts of mice through introducing Sox2, Oct3/4, c-Myc, and Klf4 under the condition of ESC culture [3]. With the ability of pluripotency and self-renewal, namely, the capacity of unlimited proliferation and differentiation into embryonic germ layers, including ectoderm, endoderm, and mesoderm, nearly all the cell types can be generated in the body from ESCs [4]. They are extensively used as ideal models *in vitro* to study the molecule mechanism of lineage-specific differentiation. Regardless of the tremendous complexity, ESCs have been successfully differentiated into various kinds of cells *in vitro* to date, such as neurons [5], cardiomyocytes [6], hematopoietic cells [7], and insulin-producing *β* cells [8]. A large number of relevant studies have provided infinite possibilities for humans to obtain specific cell types, new theories of developmental biology, and driving forces for advancing regenerative medicine.

**Figure 1:**
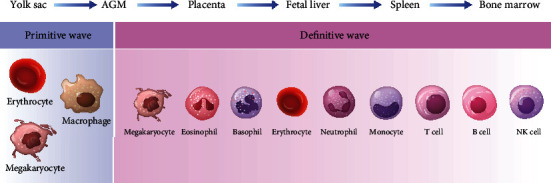
Profile of the sites, timing, and emergence of various blood cells during hematopoietic ontogeny.

However, the differentiation and development process of PSCs remains largely unknown and controversial. Hematopoietic sites are regularly changed by precise regulation *in vivo* (Figure 1). During embryogenesis, the yolk sac, aorta-gonad-mesonephros (AGM) region, fetal liver, and bone marrow (BM) are successively anatomical sites of hematopoiesis in sequence. Besides, the placenta has been reported to be an additional site that involves during the AGM to fetal liver period [9]. Based on time, hematopoiesis can be divided into two waves, each of which is temporally and spatially restricted and produces specific blood cells [10]. Known as “primitive,” the first wave generates a transitory population of hematopoietic cells in the yolk sac, including erythrocytes and some myeloid cells such as macrophages and megakaryocyte progenitors [11]. Subsequently, the “primitive” wave is promptly replaced by the “definitive” wave, also called the adult-type hematopoiesis, which causes all mature blood lineages (erythroid, myeloid, and lymphoid) to start in the AGM region, then fetal liver, and finally, BM. It is worth noting that hematopoietic stem cells (HSCs) exhibit different characteristics at different sites. For example, HSCs are cyclic in the fetal liver, while adult HSCs in the BM remain quiescent for a prolonged period [12].

Recent years have seen an explosion in the therapeutic advance of hematologic malignancies like chemotherapy and radiotherapy, which can provide limited relief with an undefined prognosis [13–15]. With few side effects, HSC transplantation is currently the most effective way to cure hematologic malignancies and autoimmune diseases [16]. Somatic HSCs mainly come from BM, peripheral blood (PB) as well as umbilical cord blood (UCB), but the number for clinical treatment is far from enough. What is more, HSCs are restricted in clinical applications because of immunological incompatibility, though they have been proven to have therapeutic efficacy. Patients may still be very likely to develop graft-versus-host disease (GVHD) after receiving HSC transplantation, which often results in death [17, 18]. Hence, there is an urgent need to find alternative sources to generate sufficient HSCs *in vitro* without incompatibility to meet clinical needs. Given the infinite differentiation potential of PSCs, it is an optimal approach to study the hematopoietic differentiation.

## 2. Hematopoietic Differentiation *In Vitro*

**Figure 2:**
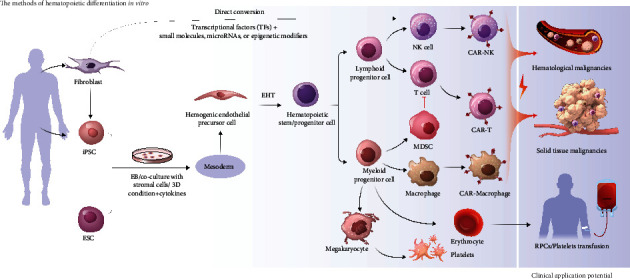
Overview of the current approaches for hematopoietic stem cells differentiation from pluripotent stem cells *in vitro* and the potential of downstream cells for future clinical application. EHT: endothelial to hematopoietic transition.

*In vitro* differentiation of PSCs is an ideal tool for studying lineage specification and commitment. Generally, hematopoietic differentiation undergoes a sequential series of cell-fate decisions with the emergence of relevant signs: the appearance of mesoderm, the specification of lateral mesoderm, the production of hemogenic endothelial precursor cells (HEPs), and endothelial to hematopoietic transition (EHT) [19]. Tied with each other to the fullest, these stages contribute to the differentiation of PSCs. At present, several differentiation protocols have been well developed to derive hematopoietic cells from PSCs (Figure 2).

### 2.1. Hematopoietic Differentiation through the Formation of Embryoid Bodies (EBs)

Spontaneously formed by PSCs, comprising epiblast cells in the inner layer and extraembryonic primitive endoderm cells in the outer layer, EBs are generated at a normally scheduled passage by plating PSCs into nontissue culture-treated plates to prevent attachment [20]. Like embryo development, PSCs can develop into structures of endoderm, ectoderm, and mesoderm under appropriate conditions as time goes on [21, 22]. The particularity of the structure, namely, multicellular three-dimensional (3D) structure, enables EBs with further cell-cell connection, communication, and substance exchange. Therefore, EBs have been widely utilized due to their irreplaceable structural and functional merits for decades as the differentiation models of PSCs by adding inhibitors, small molecules, and other growth factors to generate specific cell types [23]. Up to now, several different approaches have been taken to form EBs. It is found that an optimal procedure of EB formation can contribute to high-quality and prominent differentiation. The most frequently used approach is to remove PSCs from a self-renewing maintaining culture medium and culture them directly in a differentiation medium without a feeder layer. Then, PSCs can suspend in the medium and generate EBs composed of differentiated cells. Simple and convenient as the method is, EBs differ from each other in the morphological structure, which may reduce the differentiation efficiency of acquiring enough target cells.

### 2.2. Hematopoietic Differentiation by Coculturing with Stromal Cells

The second method of differentiating PSCs is to coculture with stromal cells such as S17, MS-5, and OP9 [24–26]. The OP9 stromal cell line was firstly established by Nakano from newborn op/op mouse calvaria in 1994, which is the most widely used stromal cell line. With a mutation in the coding area of the macrophage colony-stimulating factor (M-CSF) gene, the OP9 stromal cell line cannot express the functional M-CSF and therefore cannot promote the differentiation of PSCs into macrophages [27]. However, OP9 stromal cells can promote the hematopoietic differentiation of PSCs into erythrocytes, granulocytes, macrophages, megakaryocytes, B-lineage cells, and other types of blood cells. With the continuous improvement of methods, differentiation efficiency can be enhanced through the addition of stem cell factor (SCF), bone morphogenetic protein 4 (BMP4) [28, 29], FMS-related tyrosine kinase 3 ligand (FLT3L), and other cocktails of factors. OP9 stromal cells are commonly utilized in the differentiation of PSCs into HSCs, but few studies have disclosed the molecular mechanism that gets involved in the process of regulation, like the secretion of proteins during hematopoietic differentiation. Previously, OP9 stromal cells were used by Ueno to identify crucial genes that can support the culturing of HSCs *in vitro*. The results showed that mKirre encoding type Ia membrane protein as a mammalian homolog of Drosophila melanogaster's gene kirre was of importance to maintain hematopoiesis mainly by acting directly on HSCs [30]. In 2015, Figueiredo used proteomics analysis to identify a total of 83 proteins that were secreted from OP9 stromal cells. These proteins got involved in the development of hematopoietic and endothelial cells and imposed a great impact on the process of hematopoietic differentiation [31]. The above-mentioned reports have deeply studied the impact of OP9 stromal cells on the hematopoietic differentiation of PSCs, and a great deal of work should be done to clarify the mechanism of PSCs in the future.

### 2.3. Hematopoietic Differentiation by Direct Conversion

Direct conversion is a promising approach for cell reprogramming that allows the cell fate of interest to be obtained while bypassing an intermediate pluripotent phase [32]. It can be achieved by a specific cocktail of related transcriptional factors (TFs), small molecules, microRNAs, and epigenetic modifiers. Xie et al. (2004) indicated that macrophages could be induced from B lymphocytes by overexpression of C/EBP*α* [33]. Two years later, T lymphocytes were also found to convert into functional macrophages using C/EBP*α* [34]. However, these findings are limited to cell conversion between closely related lineages or common precursor cells, and the possibility of direct lineage occurring between two cell types distantly needs to be explored [35]. The same TFs, PU.1, and C/EBP*α* were combined to induce fibroblasts that are distantly related to blood cells in the cell type to become macrophage-like cells. The acquired cells were proven with macrophage-like phenotypes and functions. Analogously, Szabo et al. demonstrated that erythroid, granulocytic, megakaryocytic, and monocytic lineages with the capacity of *in vivo* engraftment from human dermal fibroblasts could be induced through ectopic expression of Oct4 combined with specific hematopoietic promoting cytokines [36]. These experiments implied that direct conversion of cells into another state could be completely achievable and become a novel alternative to cellular reprogramming for acquiring the ideal cells. This can not only avoid the limitations associated with the use of human PSCs but also may be the most promising development in the field of regenerative medicine. Thus, it may provide instructive and supportive signals for reprogramming whereas need to be examined in more depth.

## 3. Hematopoietic Differentiation *In Vivo*

**Figure 3:**
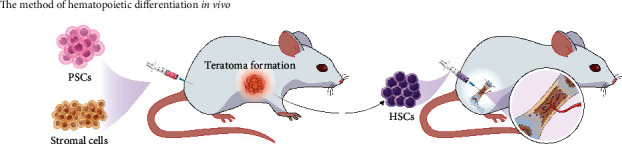
The method of hematopoietic differentiation *in vivo.*

*In vitro* generation of HSCs from PSCs is more likely to provide a new therapeutic method for replacing BM transplantation without the incidence of GVHD. However, the generation of truly functional HSCs transplantable to the recipient is quite arduous, which indicates that extrinsic niche factors are not adequate to derive HSCs from PSCs *in vitro* without essential complementary functions of intrinsic factors [37]. As an alternative, *in vivo* generation of functional HSCs from PSCs via teratoma formation has been successfully performed by some groups (Figure 3). For example, Tenen team injected iPSCs into NSG mice to produce HSPCs via teratoma formation, and generated HSPCs can be isolated from teratoma parenchyma. This suggests the ability of reconstituting a human immune system when transplanted into recipient NSG mice [38]. This is a preliminary study of *in vivo* generation of hematopoietic cells from PSCs. Similarly, Suzuki et al. combined iPSCs with OP9 cells and cytokines and then injected them into NOD/SCID mice to obtain engraftable HSCs from teratomas. The findings showed that iPSC-derived HSCs could be removed into BM from teratomas and lead to long-term reconstruction and multilineage of the hematopoietic system. Particularly, leukemia and other tumors were not found in recipients after transplantation [39]. In the subsequent years, the first-generation system was further optimized by overexpression of Gfi1b, c-Fos, and Gata2 in the process of teratoma formation, which had been previously discovered to convert fibroblasts into hematopoietic progenitors *in vitro*. This modified second generation of *in vivo* differentiation system can produce long-term functional HSCs with less time and high output compared with the first system [40]. By the ectopic expression of Lhx2 in OP9 cells, for instance, Chen et al. coinjected PSCs and OP9-Lhx2 into immunodeficient mice, which increased the proportion of hematopoietic progenitors with a transient repopulation capability compared with that of OP9/PSCs via teratoma formation. Collectively, teratoma hematopoiesis is an updated *in vivo* model for intensively studying intrinsic factors for the derivation of HSCs from PSCs.

## 4. PSCs-Derived Mature Lineage Cells in Clinical Application

As much effort has been devoted to generating HSCs from PSCs, the research on the downstream cells of HSCs has also achieved substantial progress in recent years. Next, a discussion will be held on PSC-derived blood cells, such as T cells, myeloid-derived suppressor cells (MDSCs), macrophages, NK cells, platelets, and red blood cells (RBCs), which have exhibited enormous potential in future clinical applications, especially in cancer treatment (Figure 2 and Table 1).

### 4.1. Clinical Application Potential of PSC-Derived T Cells

In recent years, a trend has appeared in cancer treatment with the rise of immunotherapy which is considered as the most promising development in cancer research and may dramatically transform the treatment of tumors. As genetically engineered T cells, chimeric antigen-receptor T cells (CAR-T cells) are used as an advanced immune treatment in clinic to direct the response to a tumor antigen [41]. As living cells are produced individually for each patient, CAR-T cells are proceeded by a complicated process, starting with the identification of patients, followed by a series of interventions aiming to collect sufficient functional T cells before treatment. After the collection of PB mononuclear cells by apheresis, CAR-T cells are generated by T cell selection and activation, lenti- or retroviral transduction with CARs, and massive expansion and finally infused back into patients [42, 43]. The curative effects have been proven in eradicating lymphoma, multiple myeloma, acute lymphoblastic leukemia, chronic lymphocytic leukemia, and other hematologic malignancies [44, 45]. In addition, solid tumors such as melanoma, sarcoma, and breast cancer hold great promise for the research and development of CAR-T cells [46]. These successful events help to catapult CAR-T cell therapy into the spotlight and make it more accessible in general clinical practice. Despite a number of relevant unresolved questions, CAR-T cell therapy is undoubtedly expected to be effective in treating more malignancies and chronic viral infections. However, new challenges have emerged with the increasing use of CAR-T cell therapy. The major obstacles to successful T cell-based immunotherapies include the limited availability and proliferative exhaustion of T cells, which are potentially overcomed by finding an infinite source of T cells [47]. The establishment of specific differentiation protocols that increase the generation of universal CAR-T cells *in vitro* may be the best option for such problem. Last year, Amelie Montel-Hagen reported that a 3D artificial thymic organoid system as a continuous culture system induced PSCs to effectively differentiate into mature and functional T cells with a diverse T cell receptor (TCR) repertoire. At the same time, it supported both hematopoietic specification and final differentiation into naïve conventional T cells CD3^+^CD8*αβ*^+^ and CD3^+^CD4^+^ [48]. Additionally, some other laboratories successfully generated T cells from iPSC with a variety of methods [49–51].

Recently, Guo et al. have identified the combinatory expression of Runx1 and Hoxa9 during the endothelial-to-hematopoietic transition process to facilitate the generation of functional T cells from PSCs according to the single-cell RNA-Seq results [52]. In detail, induced-Runx1-p2a-Hoxa9-PSCs were first constructed, and then, EB formation was applied to induce differentiation into induced hematopoietic endothelial cells (iHECs). For further hematopoietic progenitor cell maturation, the iHECs were sorted and cocultured with OP9-DL1 stromal cells. Ten days later, the generated iHPCs were transplanted into irradiated B-NDG recipient mice for T lymphocyte reconstruction. After five weeks, the donor-derived CD45.2^+^ CD3^+^ CD4^+^, or CD8^+^ iT cells were successfully detected in the spleens of recipient mice. In this study, a unique method was firstly established for generating functionally natural T cells *in vivo* from PSCs, which could provide possibilities for abundant expansion of PSC-derived T cells. However, the function of these generated iT cells requires further clarification. Therefore, the tumor antigen-specific TCR (MHC-I-restricted OVA TCR, OT1) was inserted into induced-Runx1-p2a-Hoxa9-iPSCs to evaluate the killing ability of the derived OT1 iT cells. Expectedly, the OT1 iT cells showed excellent antitumor activity in E.G7-OVA tumors model. Subsequently, Lv et al. also used this two-step protocol and transformed these iT cells together with CD19-CAR into CD19-CAR iT cells for functional assessment. As a result, these CD19-CAR iT cells exhibited outstanding performance in the eradication of B lymphoma cells (Ka539), either *in vitro* or in tumor-bearing mice [53]. Collectively, these studies have provided new ways to acquire T cells through specific systems, which demonstrate promising therapeutic potential and will be valuable tools for safe and effective T cell immunotherapy, especially combined with the innovative CAR technology. However, disease models are limited, making it necessary to carry out more experiments for further in-depth investigation.

### 4.2. Clinical Application Potential of PSC-Derived MDSCs

MDSCs are a heterogeneous group of immature immunoregulatory myeloid cells that are produced in chronic inflammatory states [54, 55]. It is increasingly proven to be a novel and crucial pattern of immune intervention with great potential for transplantation and autoimmune-related diseases. GVHD is the main barrier to the extensive use of allogeneic HSC transplantation (allo-HSCT) in the treatment of hematologic malignancies [56]. It is the result of interaction between donor T cells and recipient antigen-presenting cells (APCs) [57], which can be a devastating complication for as many as one-third of patients undergoing allo-HSCT [58]. Due to the unique functional characteristic of suppression to alloreactive T cell responses [59], MDSCs have stirred up interest among transplant immunologists in their potential clinical merits. Expectedly, researchers have demonstrated that GVHD can be efficiently prevented by MDSCs without influencing graft-versus-leukemia (GVL) effect in murine models [60, 61]. Evidence confirming that MDSCs are of vital importance for transplantation has been also shown in transiently prolonging HSC [62], corneal [63], and islet [64] graft survival. These studies emphasize that MDSCs play a significant role in downregulating immune responses, which possibly provide adequate therapeutic opportunities in various immune pathologic settings. However, efforts to generate MDSC-based treatment strategies for potential clinical applications are hindered by no reliable sources of MDSCs: MDSCs are rare in healthy donors or because of safety concerns about the use of MDSCs obtained from tumor hosts. PSC may be an ideal source for a number of MDSCs. Therefore, Zhou et al. developed a three-step differentiation approach, an efficient and credible system, in which MDSCs with active functions can be derived from the ESCs of mice. First of all, inducible HoxB4-ESs were induced to go through the EB formation process. Six days later, EB-derived cells were plated onto OP9 cells with the addition of multiple cytokine combinations, including recombinant KL, IL-6, IL-3, TPO, VEGF, Flt-3L, and M-CSF, for further culture up to 12 days. From day 6 to day 10, numerous CD115 and Ly-6C positive cells could be detected in the floating and loosely attached cells. To evaluate the suppressive activity of mESC-MDSCs *in vivo*, allo-HSCT-associated GVHD models were utilized to find that transferring mESC-derived MDSCs in an adoptive manner was effective in hampering alloreactive fatal GVHD with a high rate of long-term survival [61]. Joyce et al. established another successful system, and the acquired iPSC-derived MDSCs even comprised up to 80% CD11b^+^CD11c^low/−^ and 20% CD11b^+^CD11c^high^ with a monocyte-like morphology. It can be seen that they are capable of inhibiting T cell response and diminishing ALT leakage and CD8^+^ T cell infiltration in a murine model with autoimmune hepatitis [65]. As a whole, in the presence of several stable approaches to generate MDSCs *in vitro*, these findings may provide a reference for the clinical or commercial use of PSC-derived MDSCs. Although the results of these studies are inspiring to us, the detailed molecular modulation mechanism of protective effects is still confusing, and the adverse aspects in inhibiting host innate and adaptive immunity of cancer patients should be balanced before clinical application.

### 4.3. Clinical Application Potential of PSC-Derived Macrophages

As an ingredient of the hematologic system, macrophages not only contribute to maintaining homeostasis throughout development and adult life but also participate in the pathogenesis of inflammation, cancer, metabolic disorders, innate and adaptive immunity, and many other diseases [66, 67]. Some studies have indicated that macrophages may be feasible to mediate tumoricidal effects, thus contributing to developing macrophage-based anticancer treatments [68]. It is possible that patients will acquire great clinical benefits if owning a sufficient number of macrophages producing potent anticancer effects. Thus far, several groups have established lots of ways to derive macrophages from human or mouse iPSCs [69, 70]. Senju et al. suggested that the generated iPSCs expressing single-chain antibody (scFv) specific to human CD20 and then differentiated into iPSCs-macrophages in the presence of M-CSF and GM-CSF have the function of resisting BALL-1 cells and B cell leukemia cells *in vivo* and *in vitro*. In addition to tumor cells, this study also detected the phagocytic activity of iPSCs-macrophages in nontumor disease, which expands the scope of iPSCs-macrophages used in clinic. The accumulation of amyloid *β* (A*β*) in the brain is the main culprit of Alzheimer's disease. Hence, iPSCs were transfected with A*β*-specific scFv to observe the phagocytosis, and the scFv-transfectant iPSCs-macrophages were found to present efficient A*β*-specific phagocytosis activity.

However, the use of feeder cells and serum-containing media may lead to contamination in the clinical application. Therefore, a new differentiation culture method was proposed to generate iPSCs-macrophages under the xeno-free condition. Unfortunately, this method did not apply to all the iPSC cell clones that produced iPSCs-macrophages when performed in the conventional protocol: five iPSC cell clones were detected, and three of them died during the process of differentiation. The results indicated that the method needs to be improved efficiently and safely for further application [70].

Moreover, since macrophages often infiltrate in solid tumors, they can be particularly suitable for trafficking and surviving therein. Therefore, Senju and his colleagues continued to explore the effects of iPSCs-macrophages in different solid tumors. They reported that IFN*β*-secreting iPSCs-macrophages have a remarkable inhibitory effect on the proliferation of human pancreatic and gastric cancers implanted in the peritoneal cavity of mice [71]. In short, it is proven that genetically modified iPSCs-macrophages have the potential of therapeutic application to Alzheimer's disease, blood cancers, and solid cancers.

As mentioned above, CAR-T cell therapy demonstrates excellent performance in the treatment of hematologic malignancies, but its application in solid tumors is challenging, mainly because T cells have difficulty penetrating and surviving in the TME [72]. The phagocytosis activity of iPSCs-macrophages against solid tumor cells has been mentioned in the last paragraph, but how effective when they combined with CARs? Excitingly, Klichinsky et al. designed macrophages to express CARs targeting their phagocytic activity towards tumor cells. Such CAR macrophages (CAR-Ms) exhibited antigen-specific phagocytosis and tumor clearance *in vitro* and prolonged the overall survival of mouse ovarian cancer models [73]. Analogously, we have also worked with other research groups to develop CAR-expressing iPSC-induced macrophages, showing excellent performance on antitumor effects *in vitro* and *in vivo* [74]. Overall, more data *in vivo* are needed before clinical application, and the immense value will be beyond imagination if a large number of macrophages can be obtained from PSCs.

### 4.4. Clinical Application Potential of PSC-Derived NK Cells

Natural killer (NK) cells, first identified as uniquely innate lymphocytes in 1975, show cytotoxicity against certain target cells without the need for specific antigen recognition [75–77]. The activation of NK cells depends on the balance between activating and inhibitory signals from receptors to distinguish healthy cells from infected or tumor targets [78–81]. Due to certain innate properties, NK cells are considered to be a promising clinical utilization in human immunotherapies against various malignancies. Importantly, the occurrence of GVHD is completely avoided compared to T cells, and NK cells are supplementary for T cell therapy to some extent, which may have the ability to resolve some limitations of T cell therapy. For NK cell immunotherapy, however, it is critical to obtain a sufficient number of safe, pure, and functional NK cells. Existing studies have shown that NK cells can be produced from various sources, including bone marrow, umbilical cord blood (UCB), and peripheral blood (PB) [78]. Nevertheless, UCB-NK cells and PB-NK cells are a heterogeneous mixture of NK cells and other immune cells that are variant between individuals. What is more, it has also been proven to be both safe and effective in multiple clinical studies of allogeneic NK cells therapy [82–85]. Like other blood cells, NK cells can be derived from PSCs as well. In the past several years, a series of studies have advanced the development of mature NK cells from hPSCs. The progress of PSC-derived NK cells in recent years is summarized in this part as well as their potential translational application in the clinic. Woll et al. sorted CD34^+^ or CD34^+^CD45^+^ hematopoietic progenitors from H9/S17 cells (H9 hESCs differentiate on S17 stromal cells) and cultured them on AFT024 cells in medium with the addition of IL-15, IL-3, IL-7, SCF, and Flt-3L for a period of time. The findings indicated that functional NK cells could be efficiently generated from hESCs, with the expression of CD94/NKG2a and KIRs, which are proven to have the ability of lysing human tumor cells by both antibody-dependent cellular cytotoxicity (ADCC) and direct cell-mediated cytotoxicity. More importantly, the production of IFN-*γ* can be achieved when hESC-derived NK cells were stimulated with IL-12 and IL-18 [86]. Last year, Kaufman and Zhu modified their previous method to finally produce mature, functional NK cells with higher efficiency and less time (it usually takes one month to acquire >90% CD45^+^CD56^+^ cells from most of iPSC or ES lines through EBs differentiation). In this way, the rapid generation of an essentially unlimited number of homogenous NK cells enables it to be applied in targeted, standardized immunotherapy for treating infectious diseases and refractory cancers [87]. Matsubara and his colleagues also established a differentiation protocol for the development of functional NK cells from hPSCs, which exhibited tremendous performance in struggling against K562 tumor cells *in vitro* and *in vivo* [88]. Compared with other methods, the main advantage is that hPSCs can be differentiated under completely chemically defined conditions in two-dimensional culture, which do not contain xeno-derived stromal cells like OP9.

Like CAR-T cells and CAR-macrophages, CAR-NK cells have been also used against diverse malignancies. To date, CAR-NK cell therapy has been proven to kill solid and hematological tumor cells in preclinical and clinical trials, demonstrating its potential as a ready-made product with wide clinical applications [89]. It eliminates tumors not only by NK cell receptors themselves but also by the ability of CARs to specifically recognize antigen-expressing tumors [90]. Thus, combining novel CAR technology with the optimization of developing NK cells from PSCs provides reasonable expectations for the future. Moreover, due to potentially broad applications in the treatment of various human malignancies, PSCs provide new solutions to obstacles relating to traditional NK therapies in banking, manufacturing, and improving antitumor activities.

### 4.5. Clinical Application Potential of PSC-Derived Platelets

As the smallest anucleate cells (2-4 *μ*m in diameter), platelets are released from megakaryocytes in the bone marrow. They are indispensable in the process of neoangiogenesis, innate immunity, and inflammation, especially in hemostasis and thrombosis [91–95]. In clinic, chemotherapy or radiotherapy, massive hemorrhage, genetic platelet disorders, and other events may lead to reduction and/or dysfunction of platelets, namely, thrombocytopenia and/or platelet dysfunction. Platelet transfusion is the optimal way for patients suffering from these bleeding complications. However, it still has limitations and warning points: (1) platelets for clinic are far from sufficient, due to not only ascending demands but also lacking blood donors; (2) platelets derived from donors may contain pathogen contamination, which cannot be timely detected under the so-called window period of infection; (3) the limited shelf life of platelets is only 4-5 days due to their short activity of function; (4) the suitable storage condition at room temperature is in favor of bacterial reproduction, enhancing the risk of pathogen contamination to some extent [96–98]. Because of these issues, there is a real need to make more efforts to develop strategies for generating effective and efficient, nondonor-dependent systems *in vitro* to continuously provide platelets for clinical use. Hence, the generation of megakaryocytes or platelets from PSCs would be an ideal strategy for eliminating donor dependency and insufficient quantity.

The first study of using hESCs to generate megakaryocytes was reported in 2006. In this study, the authors established a method of producing megakaryocytes from hESCs, and these hESC-derived megakaryocytes expressed von Willebrand factor in the cytoplasm, CD41a, and CD42b on their surface and exhibited the presence of DNA ploidy distribution, which are characteristics of megakaryocyte lineage cells. However, the limitation is that they just detected some classic markers of megakaryocytes without further functional experiments [99]. In 2008, Takayama et al. developed a novel system that enables hESCs to efficiently differentiate into platelet-forming megakaryocytes. They found that balloon-like structures named ESC-derived sacs could be formed through coculturing ESCs with OP9 or C3H10T1/2 stromal cells, which are considered to be the beneficial microenvironment for the generation of hematopoietic progenitors. Then, a large number of mature megakaryocytes could be produced from the hematopoietic progenitors within ESC-derived sacs, which were able to release platelets that could be activated by ADP or thrombin. Compared with the numbers *in vivo*, however, fewer platelets were generated in this system, which may be due to the lack of some thrombopoiesis stimuli under this *in vitro* environment [100]. Admittedly, great progress has been made in this field over the last ten years. In 2014, Feng et al. succeeded in differentiating hESCs/iPSCs directly into megakaryocytes and platelets under a serum-free, feeder-free condition. Particularly, instead of using the method of EB formation, they used a hemogenic endothelium intermediate to avoid inconsistencies and low yields of megakaryocyte progenitors. And the conventional functions of these achieved megakaryocytes and platelets were successfully verified *in vitro* and *in vivo*, respectively. More importantly, this method also allowed cryopreservation of megakaryocyte progenitors in case of an urgent need for numerous platelets [101]. Recently, Zhang et al. provided an applicable protocol for the production of platelets. A polystyrene CellSTACK culture chamber was used to modify the 2D differentiation environment into a 3D sphere-like differentiation environment for megakaryocytes during the whole differentiation process. The generated megakaryocytes with obvious characteristics had the function of cytokines synthesized and secreted as well as produced platelets that could be activated by thrombin or fibrinogen. The breakthrough of this experiment is that by combining novel 3D culture technology with previous experience, the induction system is completely xeno-free, feeder-free, or transgene-free for the generation of megakaryocytes, which is better for future clinical applications [102]. These results indicated the progress in the large-scale generation of PSC-derived megakaryocytes and platelets as well as possible therapeutic applications in clinical settings.

### 4.6. Clinical Application Potential of PSC-Derived Erythroid Cells

Like platelets, red blood cells (RBCs) are one of the important blood ingredients used in transfusion medicine. RBC transfusion is an indispensable cell therapy for many patients with massive blood loss, anemia, or various hematological disorders [103]. However, the supply of RBCs remains dependent on healthy voluntary donations and the security of RBCs transfusion has become an important medical concern in most countries to date. Advances in recent years have increased the likelihood of therapeutic production of RBCs *in vitro*. Even so, the production of large-scale RBCs required for transfusion is still a major challenge.

Over the past few years, many scientists have concentrated on the induction of functionally mature RBCs from hESCs or iPSCs for application in therapeutic transfusion. For the first time, Lapillionne et al. reported the complete differentiation of human iPSCs into definitive erythrocytes that can mature to enucleated red blood cells in 2010 [104]. The adult skin primary fibroblast lines and human fetal fibroblasts (IMR90) were used to construct iPSCs firstly and proceeded with the following two steps: (1) differentiation of iPSCs to obtain early erythroid commitment by means of EBs formation in the medium containing cytokines and human plasma for 20 days; (2) maturation to the stage of cultured RBCs supplement with cytokines. At the end of culture, CD235a and CD71 could be strongly detected. The findings seemed to be promising, but the generated RBCs expressed mostly fetal hemoglobin (hemoglobin F), which is slightly less efficient for oxygen delivery than adult hemoglobin.

In 2019, Bernecker et al. described a simplified, feeder-free, and xeno-free culture system for prolonged RBC generation from hiPSCs with three kinds of cytokines: SCF, EPO, and IL-3 [105]. After EB formation in low-attachment plates for 5 days, EBs were transferred into adherent plates for hematopoietic differentiation. Two weeks later, the hematopoietic cell forming complex (HCFC) as an adherent cellular complex was formed, which consisted of blistered spheroids with “red islands” surrounded by a network of stromal cells. Single cells were continuously released from the HCFC in 3-8 weeks. Ultimately, cells were harvested from the supernatant and differentiated into RBCs in a three-phase erythropoiesis system for 18 days [106]. By contrast, this system has the following merits: (1) lower labor costs; (2) less use of cytokines; (3) continuous collection of hematopoietic cells for up to 8 weeks; (4) homogeneous maturation through all the human erythropoiesis stages such as the progenitor cell stage; (5) increased enucleation rate (mean 40%, up to 60%) compared to the published data (10% and 20%). These studies have provided more ways of differentiation into mature RBCs to establish an efficient, reliable protocol for RBC generation to promote the development of RBC clinical treatment.

## 5. Current Challenges regarding HSCs and Downstream Cells Generated from PSCs

To date, PSC-derived cellular therapy has great therapeutic potential with an extensive range of applications from correcting hematologic disorders to regenerative demands [107]. Since a variety of diseases have been modeled under *in vitro* conditions, the generation of HSCs and downstream cells from PSCs has been considered as a promising approach to facilitate the further development of related disease therapies. Unfortunately, hematopoietic differentiation *in vitro* still has certain limitations that impede its application in clinical settings.

Hematopoietic stem cell transplantation (HSCT) is currently one of the optimal treatment choices for various malignant and nonmalignant hematological disorders. Hematopoietic stem cells are infused via intravenous injection to be transplanted into patients, which is based on the ability of donor HSCs to migrate and localize into recipient BM niches. Then, they begin to proliferate and expand. Finally, they will differentiate and give rise to mature cells [108]. It is worth noting that not all the transplanted cells succeed in reaching the BM microenvironment but only with a small fraction of infused HSCs (10%) [109]. This process, known as “homing,” directs the migration of stem cells through different signaling pathways which are mediated by growth factors or chemokines binding to corresponding receptors expressed on stem cells, thereby guiding stem cells to a particular target [110]. Thus, increasing the homing capacity of stem and progenitor cells is a crucial way to improve transplantation. Although HSCT failure is becoming rare, it is still a tricky problem to call for definite answers, at least for the moment, due to the not fully elucidated mechanism and the incomplete ability to modulate it.

In addition to the ongoing challenges of differentiating hPSCs into HSCs that are capable of long-term engraftment, it is necessary to determine the safety of generated blood cells. Accurate control of epigenetic changes in the process of hematopoietic differentiation plays a crucial role in ensuring the safety of generated cells for clinical use [111]. It has been reported that hematological malignancies can be triggered by the abnormal control of gene expression in HSCs derived from PSCs [112]. For example, MLL-AF4 engineering has been demonstrated for possible leukemia transformation of long-term engrafted iPSC-HSCs [113]. Dysregulation of HOX genes is closely related to acute lymphoid leukemia and acute myeloid leukemia [114]. EZH2 overexpression or downregulation can lead to tumorigenesis [115]. Therefore, safety evaluation is required for the generation of therapeutic-grade HSCs from PSCs to avoid possible carcinogenesis. Similarly, PSC-derived downstream cells are also in possession of the aforementioned problems.

Many studies have shown the effective differentiation of hPSCs into various blood cells, but the majority has not yet addressed the problem of large-scale cell generation. For example, RBC therapy is particularly challenging with the demand of receiving at least 1 × 10^12^ cells per transfusion unit per patient. The demands of target cells and stromal cells cannot be completed by using conventional time- and labor-consuming methods, which would require a bioreactor system to maintain cells. In terms of bioprocessing, cells with a density of 1 × 10^8^ cells per mL can be obtained for the purpose of expanding large-scale manufacturing. Due to the use of expensive cytokines in the differentiation stages, what it requires is a very high cost. According to the report, it is predicted that the cost of RBCs generated from hPSCs is between 8000 and 15,000 USD per transfusion unit [116]. Therefore, cost-effective and highly easy-to-use strategies should be devised to make important progress towards large-scale cell production for future clinical applications.

The use of feeder cells and serum is more likely to strengthen maturation and optimize cell population expansion; however, the existence of foreign materials will bring about the risk of contamination and impose necessary restrictions on clinical applications. Therefore, to achieve efficient expansion and differentiation of hPSCs, it is sought to replace feeder/serum conditions with defined culture conditions and small molecules, thus making them suitable as master cells for good manufacturing practice (GMP)-grade production in the future.

For the function of derived cells, the greatest challenge is to find effective ways to simulate the internal environment and acquire functional blood cells *in vitro*. The homeostasis of hematopoietic differentiation occurring at different sites is controlled by the precise regulation of the microenvironment, which cannot be easily imitated *in vitro*. This difficulty will lead to functional defects in the cells generated from PSCs, which need to be further enhanced. Through most of the current protocols, both hESCs and hiPSCs seem to have the ability to generate blood cells with a definitive phenotype and express the proper surface marker for functional verification. However, hiPSCs can be manipulated more unrestricted without ethical problems. More animal models are needed to be established for further evaluation of differences in the generated cells from hESCs and hiPSCs, including but not limited by critical gene expression, morphology, and function.

Other issues, such as the failure to acquire all types of blood cells and unstable differentiation efficiency, are the unsolved problems as well. To sum up, it seems that PSC-derived hematopoietic stem/progenitor cells and downstream cells need to be questioned about the suitability in clinical applications.

## 6. Advanced Technologies in PSC Differentiation

### 6.1. Heterogeneity of Cell Populations Identified by Single-Cell Analysis during Differentiation

Although several methods of differentiating PSCs *in vitro* are well-established, the genetic mechanism underlying differentiation remains poorly characterized. A promising tool named single-cell transcriptomics has emerged in recent years. Single-cell sequencing is conducive to investigating the heterogeneity of different cell populations during differentiation, discovering new types of cells, and inferring putative differentiation routes [117]. In combination with this new method, Yang et al. demonstrated that c-Kit^+^/CD41^+^ EMP-like progenitors cultured *in vitro* on day 6 were similar in composition to E9.5 YS EMPs *in vivo*, including an erythroid, myeloid/innate immune population and a residual hemogenic endothelium-like population. This suggested that ESC differentiation *in vitro* was of great relevance to the sequential developmental stages of hematopoiesis *in vivo* [118]. As a stage of hematopoietic development, EHT plays a pivotal role in the generation of hematopoietic cells. Through transcriptome analysis, Angelos et al. showed that hESC-derived hematopoietic endothelial cells and hematopoetic progenitors shared a common developmental pathway, while nonhematopoietic endothelial cells exhibited heterogeneity. Hence, the findings provided a novel strategy to optimize the production of hematopoietic cells from hPSCs [119]. Lately, Guo et al. used single-cell transcriptome and functional analysis to illustrate the cellular trajectory of PSC-induced T lineage. The results unveiled the specification of T lineage early determined at the stage of hemogenic endothelial cells and identified bona fide prethymic progenitors [52].

In summary, these above-mentioned reports highlighted the heterogeneity of different cell populations during hematopoietic differentiation *in vitro*. However, further analysis remains to be carried out to investigate the underlying mechanism and provide further insights into the key molecular drivers that regulate the development of blood cells from PSCs *in vitro*.

### 6.2. Simulating Microenvironment In Vivo, 3D Biomaterials Are Beneficial to Hematopoietic Differentiation

Bone marrow niches (microenvironment) constitute a complex, multidimensional internal system, involving both physiochemical (O_2_ concentration, stiffness, and extracellular matrix presentation) and biochemical (cells, cytokines and growth factors) factors that determine the cell fate of HSCs [120]. Balancing the maintenance and differentiation of HSCs enables themselves to well interact with the microenvironment in the human bone marrow and ensure life-long blood cell production. Though the hematopoietic differentiation of PSCs is currently mature and stable by means of OP9 and EB, there is also the same disadvantage: PSCs are mostly cultured in a 2D environment that is overly simplistic culture substrate and different from the internal environment. This fails to completely replicate the niches or microenvironments where cells live, including intercellular functions and signaling pathways occurring naturally *in vivo* [121]. During embryonic development, cells are interactive and surrounded by a 3D extracellular matrix that consists of laminin, collagen, and other proteins intertwined with proteoglycan [122]. Capable of activating downstream pathways to realize the regulation of specific gene expression, these interactions are required by proper tissue development. To sum up, 2D culture has the major problem of limited structure, which is certainly not equal to the internal tissue considering the complexity of the cellular microenvironment [123]. Thus, it is pressing to establish *in vitro* niches which mimic the natural development of HSCs from PSCs. Recently, our group has successfully established 3D self-assembling peptide hydrogel containing hematopoietic cytokines to promote the differentiation of mPSCs into clusters of hematopoietic cells [124]. It is concluded that 3D self-assembling peptide hydrogel can provide a 3D environment for *in vitro* hematopoietic differentiation of mPSCs by evaluating the function of hematopoietic cells derived from a 3D system with related hematopoietic TFs, makers, CFUs, and lymphoid differentiation potential. In addition, hematopoietic cells obtained from the 3D system have potential in short-term hematopoietic reconstitution [125]. Li and his colleagues also identified similar advances that an artificial matrix composed of designer 3D self-assembling peptides can enhance the formation of EBs and provide cues favoring the neuronal differentiation of ESCs [126]. These results manifested the superiority of 3D biomaterials as an advanced technology for cell development. Thus, it would be the revolutionary approach for the fast and reliable generation of HSCs *in vitro* and satisfy the growing demand for stable, clinical-grade HSC transplantation. There is still additional research to be performed to make the intricate, complex platform as similar as the BM niche.

## 7. Summary

The advent of PSCs has contributed to understanding the early stage of hematopoietic development in the mammalian and broadening and deepening the human cognition of disease progression. However, several issues remain to be solved due to the limited insights into the generation of various blood cells *in vitro*. This review provides an overview of the possibilities and challenges regarding the production and use of PSC-derived blood cells.

With the increasing incidence of hematological malignancies, PSCs may become potent sources for the generation of numerous T cells that can be manufactured into universal and off-the-shelf cell products to facilitate the novel CAR-T cell therapy. Certainly, PSC-derived MDSCs, macrophages, NK cells, platelets, and red blood cells exhibit promising performance in the treatment of refractory diseases like GVHD, anemia, and some solid tumors, even some nonhematologic diseases. The findings of current studies have offered clinical information on the future treatment of various diseases. Furthermore, progress in other technologies, such as CRISPR/Cas9, 3D cell culture, and organoid switches, will accelerate the pace of PSC-specific differentiation and therapeutic development. To better take advantage of PSC-derived cells, more work remains to be done before clinical application. In the next few years, it is expected to solve the existing problems of in vitro hematopoietic differentiation and the generation of functional immune cells from PSCs.

## Figures and Tables

**Table 1:** Selected reports for the functional blood cells derived from PSCs.

Cell source	Methods	Factors	Generated cells	Specific markers	Production efficiency	Functional assessment	Reference
Mouse (MHC-I-restricted OVA TCR, OT1)-inducible Runx1-p2a-Hoxa9-induced pluripotent stem cells	EB formation and coculture with OP9-DL1 for OT1-iHPC generation and T lymphocyte reconstitution *in vivo*	BMP-4, VEGF, AFT024-mIL3, AFT024-mIL6, AFT024-mSCF, AFT024-mFlt-3L, iron-saturated transferrin, monothioglycerol, ascorbic acid	OT1-iT cells	CD8	~9.1% in PB	Effectively eradicated E.G7-OVA tumor cells *in vivo*	52
Mouse pluripotent stem cells	EB formation and coculture with OP9-DL1 for iHPC generation and T lymphocyte reconstitution *in vivo* and then isolated and infected with the CD19-CAR	BMP-4, VEGF, AFT024-mIL3, AFT024-mIL6, AFT024-mSCF, AFT024-mFlt-3L, iron-saturated transferrin, monothioglycerol, ascorbic acid	CD19-CAR-iT cells	CD3, CD4, and CD8	CD3^+^CD4^+^63.2% CD3^+^CD8^+^35.3% in spleens	Efficiently eliminated B lymphoma cells Ka539 *in vivo* and *in vitro*	53
Mouse inducible HoxB4 embryonic stem cells	EB formation and coculture with OP9 stromal cells	Recombinant KL, IL-6, IL-3, TPO, VEGF, Flt-3L, and M-CSF	MDSCs	CD115, Ly-6C	53.9%	Prevented GVHD following allogeneic bone marrow transplantation	61
Mouse-induced pluripotent stem cells	Coculture with OP9 stromal cells and hepatic stellate cells	GM-CSF	MDSCs	CD11b, CD80, CD86 and B7-H1	Upto 80% CD11b^+^CD11c^low/−^and 20% CD11b^+^CD11c^high^	Regulated T cell responses *in vitro* and *in vivo* and diminished CD8+ T cell infiltration and ALT leakage in an autoimmune hepatitis murine model	65
Human-induced pluripotent stem cells Human embryonic stem cells	Coculture with OP9 stromal cells for hematopoietic differentiation and isolated CD235a/CD41a^–^CD45^+^ for myeloid progenitors generation	M-CSF, IL-1*β*	Macrophages	CD115, CD163, CD14, and CD68	35%~44%	The ability of a self-quenched conjugate of ovalbumin (DQ-OVA) uptake, processing, and allogeneic stimulation	69
Human-induced pluripotent stem cells	In earlier stage: coculture with OP9 stromal cells; in later stage: cultured in the presence of GM-CSF and M-CSF without OP9 cells	GM-CSF, M-CSF	Macrophages	CD11b, CD14 and CD68	Unmentioned	Ingested both immunoglobulin G-opsonized and unopsonized zymosan particles and showed chemotactic response to C5a. Inhibited the growth of BALL-1 cells *in vivo* and *in vitro*	70
CAR-expressing human-induced pluripotent stem cells	EB formation	BMP-4, bFGF, VEGF, SCF, IGF-1, IL-3, M-CSF, and GM-CSF	CAR-macrophages	CD11b, CD14, and CD163	~100% of CD11b and CD14 expression	Inhibited CD19-expressingK562 or leukemia cells mesothelin-expressing OVCAR3/ASPC1 ovarian/pancreatic cancer cells *in vitro* and ovarian cancer cells HO8910 *in vivo*	74
Human embryonic stem cells	Coculture with mouse bone marrow stromal cell line S17 and cocultured with AFT024 stromal cells	Ascorbic acid, 2-ME, IL-15, IL-3, IL-7, Flt-3L, and SCF	NK cells	CD56	About 30%~40% on day 28	Showed significant cytolytic activity to K562 cells *in vitro* and upregulate IFN-*γ* production in response to IL-12/IL-18 stimulation	86
Human embryonic stem cells and induced pluripotent stem cells	Spin EB formation	BMP-4, bFGF, VEGF, IL-15, IL-3, IL-7, Flt-3L, and SCF	NK cells	CD45, CD56	>90%	Killed against K562 cells and MOLM13 cells *in vitro*	87
Human embryonic stem cells and induced pluripotent stem cells	Completely chemically defined condition in two-dimensional culture	BMP-4, VEGF, IL-15, IL-7, Flt-3L, and SCF	NK cells	CD56	87.27 ± 4.52%	Killed K562 cells *in vivo* and *in vitro*	88
Human embryonic stem cells	Cocultured with C3H10T1/2 or OP-9 cells	IL-6, IL-11, SCF, TPO, and heparin	Platelets	CD41a, CD42b	Most small particles positive for CD41a and CD42b	Displayed integrin *α*IIb*β*3 activation and spreading in response to ADP or thrombin	100
Human embryonic stem cells and induced pluripotent stem cells	Serum/feeder-free conditions	BMP-4, bFGF, VEGF, TPO, SCF, Flt-3L, IL-3, IL-6, IL-9, and heparin	Platelets	CD41a, CD42b	>80%	Formed aggregates, lamellipodia, and filopodia after activation *in vitro* and thrombus *in vivo*	101
Human embryonic stem cells	EB formation in a 3D CellSTACK culture chamber	AA2P, BMP-4, bFGF, CHIR99021, VEGF, SB431542, SCF, TPO, IL-3, Flt-3L, IGF1, GM6001, and IL-11	Platelets	CD41a, CD42b, and CD61	>80% coexpressed with CD41a and CD61 and >50% coexpressed with CD41a and CD42b	Showed increased CD62P expression and clot aggregation after thrombin activation	102
Human-induced pluripotent stem cells	EB formation	SCF, TPO, FLT-3L, BMP4, VEGF, IL-3, IL-6, and EPO	RBCs	CD235a and CD71	1 × 10^6^ hiPSCs gave rise to up to 4.4 × 10^8^ mature erythroid cells	CO flash photolysis experiments confirmed that the hemoglobin F in cultured RBC is functional	104
Human-induced pluripotent stem cells	EB formation	Holotransferrin, plasma, SCF, IL-3, EPO, and insulin	RBCs	CD36 and GPA	CD36 (>90%) and GPA (96.0% ± 4%)	HPLC revealed the presence of predominantly fetal hemoglobin in hiPSC-derived RBCs	105

## Abbreviations

PSC: Pluripotent stem cell
iPSC: Induced pluripotent stem cell
ESC: Embryonic stem cell
AGM: Aorta-gonad-mesonephro
BM: Bone marrow
HSC: Hematopoietic stem cell
PB: Peripheral blood
UCB: Umbilical cord blood
HEP: Hemogenic endothelial precursor cell
EHT: Endothelial to hematopoietic transition
EB: Embryoid body
3D: Three-dimension
M-CSF: Macrophage colony-stimulating factor
SCF: Stem cell factor
BMP4: Bone morphogenetic protein 4
FLT3L: FMS-related tyrosine kinase 3 ligand
TF: Transcription factor
CFU: Colony forming unit
MDSC: Myeloid-derived suppressor cell
CAR-T cell: Chimeric antigen-receptor T cell
iT cell: Induced T cell
allo-HSCT: Allogeneic hematopoietic stem cell transplantation
APC: Antigen-presenting cell
GVL: Graft-versus-leukemia effect
TME: Tumor microenvironment
CAR-M: Chimeric antigen-receptor macrophage
EMP: Erythroid and myeloid progenitor.
